# Protect the kidneys against contrast medium: **Re: Wu J, et al. A novel contrast-induced acute kidney injury mouse model based on low-osmolar contrast medium. Ren Fail. 2022;44:1345–1355**

**DOI:** 10.1080/0886022X.2022.2130803

**Published:** 2022-10-10

**Authors:** Tadashi Yoshida

**Affiliations:** Apheresis and Dialysis Center, Keio University School of Medicine, Tokyo, Japan

Dear Editor,

I read with great interest the article entitled ‘A novel contrast-induced acute kidney injury (CIAKI) mouse model based on low-osmolar contrast medium’ by Wu et al. [1] The authors examined multiple combinations of pretreatment for the establishment of a novel, efficient, and reproducible CIAKI model in mice. They eventually found that uni-nephrectomy combined with 24 h-water deprivation and furosemide administration 20 min before the application of contrast medium is a feasible pretreatment to induce CIAKI. The study may provide a significant advance in the research field of developing novel treatment for CIAKI, but I have some comments on this study.

First, multiple parameters which are required to understand the pathophysiology of CIAKI are lacking for the study. Although the authors showed the changes in serum creatinine levels in their CIAKI model, several important parameters, such as the urine volume, body weight, water intake volume, glomerular filtration rate, and neutrophil gelatinase-associated lipocalin levels in the urine, were not measured. In addition, only the temporal changes in the levels of blood urea nitrogen, but not of serum creatinine, were shown. The authors showed that long-term water deprivation, such as 48 and 72 h, was not an appropriate pretreatment to induce CIAKI, whereas the combination of 24-h water deprivation and furosemide injection was an optimal pretreatment for inducing CIAKI in uni-nephrectomized mice. It is questionable whether dehydration itself was sufficient to successfully induce CIAKI, or furosemide injection had additional detrimental effects on the kidneys. In this regard, several additional data described above are needed to address the underlying mechanisms of this CIAKI model in detail.

Second, although the authors used male C57BL/6 mice in the study, it is interesting to determine whether the CIAKI model is also applicable to other mouse species. As many of transgenic and knockout mice are on the C57BL/6 background, the model would be very useful to perform gain-of-function and loss-of-function studies in mice in future. However, it is known that several mouse models for studying renal diseases cannot be applicable to multiple mouse species. For example, BALB/c mice are sensitive, while C57BL/6 and FVB/NJ mice are highly resistant to adriamycin nephropathy [2,3]. In addition, C57BL/6 mice are calcification resistant, whereas DBA/2 mice are susceptible to calcification [4]. If susceptibility to CIAKI is strain-specific, breeding experiments would be a potential tool to identify a gene locus that confers susceptibility to CIAKI. Further studies are needed to address this question.

CIAKI is a serious complication of the use of iodinated contrast media in diagnostic and interventional procedures, and it is one of the leading causes of hospital-acquired acute kidney injury in the world. Thus far, multiple approaches, including statin use [5], hydrogen gas inhalation [6], etc. have been proposed as potential prophylactic therapies for CIAKI. It is of great interest to examine the effects of these therapeutic approaches in the mouse CIAKI model [1]. In addition, a lot of genetically engineered mice, in which genes of interest are overexpressed or deleted, are easily available. It is also interesting to perform gain-of-function or loss-of-function experiments by using genetically modified mice on the C57BL/6 background to find out the key molecules and pathways in CIAKI. Establishment of new therapeutic strategies for CIAKI is desired by using this mouse CIAKI model [1].
